# Ovarian Vein Syndrome Presenting as Recurrent Right-Sided Flank Pain: A Case Report

**DOI:** 10.7759/cureus.102406

**Published:** 2026-01-27

**Authors:** Marwa Faisal, Sara Alshehabi, Mohammed Yusuf, Sawsan Kadhem

**Affiliations:** 1 College of Medicine, Mansoura University, Mansoura, EGY; 2 College of Medicine, First Moscow State Medical University, Moscow, RUS; 3 College of Medicine, Zhejiang University, Hangzhou, CHN; 4 Department of Radiology, Government Hospitals, Manama, BHR

**Keywords:** case report, conservative management, ct imaging, endovascular embolization, flank pain, gonadal vein, hydronephrosis, ovarian vein syndrome, ureteral obstruction, urinary tract infection

## Abstract

Ovarian vein syndrome (OVS) is a rare cause of ureteral obstruction resulting from extrinsic compression by a dilated ovarian vein, most commonly affecting women of reproductive age. Its nonspecific presentation, including intermittent flank or abdominal pain, urinary symptoms, and recurrent UTIs, often mimics more common conditions such as ureterolithiasis, appendicitis, or gynecologic pathology, leading to delayed diagnosis. Imaging modalities, particularly contrast-enhanced CT, are essential for identifying dilated ovarian veins compressing the ureter and associated hydronephrosis. We report a case of a woman with recurrent right-sided abdominal and flank pain initially attributed to physiologic hydronephrosis during pregnancy, whose diagnosis of OVS was established years later after longitudinal imaging revealed a markedly dilated right gonadal vein causing ureteral compression. Management of OVS is individualized and may include conservative measures such as analgesia, hydration, and monitoring of renal function, or interventions such as endovascular embolization or surgical ligation in cases of persistent or progressive obstruction. This case highlights the importance of considering OVS in the differential diagnosis of unexplained unilateral hydronephrosis and recurrent flank pain, underscores the role of imaging in timely diagnosis, and demonstrates that conservative management can be effective in selected patients.

## Introduction

Ovarian vein syndrome (OVS) is a rare clinical entity characterized by ureteral obstruction secondary to dilatation or thrombosis of the ovarian vein [1,2]. First described in the mid-20th century, it is most commonly observed in multiparous women and is often associated with chronic pelvic congestion, previous pregnancy, or pelvic surgery [1,3]. The syndrome may present with nonspecific symptoms, including intermittent flank or abdominal pain, hematuria, or UTIs, which can mimic more common conditions such as ureterolithiasis, appendicitis, or gynecologic pathology [1-3]. Because of its rarity and subtle radiologic findings, OVS is frequently underdiagnosed, and patients may experience recurrent symptoms over the years before the correct diagnosis is established [1-3]. Imaging modalities such as ultrasound, CT, MRI, and venography are critical for identifying dilated ovarian veins and assessing their relationship to the ureter [3,4].

Management of OVS remains individualized, ranging from conservative observation to surgical or endovascular intervention [5,6]. Minimally invasive procedures, including laparoscopic ovarian vein ligation or interventional radiology-guided embolization, have emerged as effective options in patients with persistent symptoms or significant obstruction [5,6]. Conservative management, including analgesia and hydration, may be appropriate in patients with mild or intermittent symptoms and stable renal function [1-4]. Given its uncommon nature, case reports and longitudinal observations provide valuable insight into the clinical course, diagnostic challenges, and treatment considerations for this syndrome. Reporting such cases enhances awareness among clinicians and underscores the importance of considering OVS in the differential diagnosis of recurrent right-sided abdominal or flank pain.

## Case presentation

A female patient of reproductive age experienced recurrent episodes of right-sided abdominal and flank pain over a prolonged period. Her initial presentation occurred during pregnancy, when she presented to the emergency department with acute right lower abdominal pain concerning for appendicitis. She denied fever, nausea, vomiting, urinary symptoms, or changes in bowel habits. Her obstetric history was otherwise unremarkable, and the pregnancy had been progressing normally. On physical examination, she was hemodynamically stable. Abdominal examination revealed localized tenderness in the right lower quadrant without guarding, rebound tenderness, or signs of peritonitis. There was no costovertebral angle tenderness at that time.

**Figure 1:**
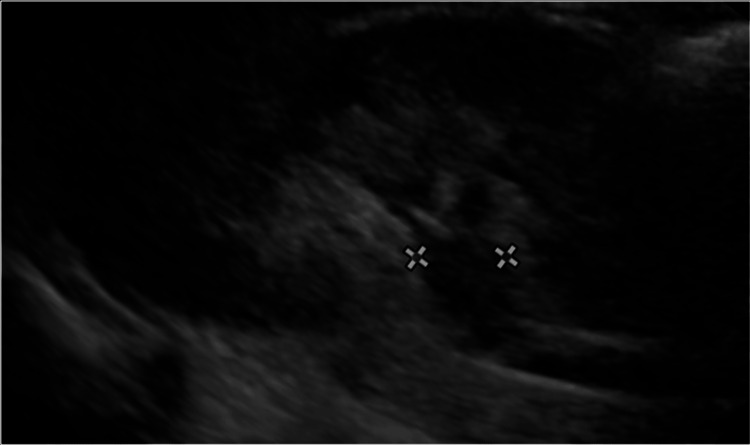
Grayscale ultrasound of the right kidney showing hydronephrosis Grayscale ultrasound image of the right kidney demonstrates a dilated renal pelvis consistent with hydronephrosis. The renal parenchyma appears normal, and no ureteral calculi are visualized.

Initial laboratory investigations demonstrated a normal white blood cell count and no elevation in inflammatory markers. Urinalysis was unremarkable, with no evidence of infection or hematuria. An abdominal ultrasound was performed to evaluate for appendicitis and obstetric pathology. The appendix was not visualized, and there were no adnexal or uterine abnormalities identified. However, the ultrasound incidentally demonstrated mild right-sided hydronephrosis, with an anteroposterior diameter of the renal pelvis measuring approximately 13 mm (Figure 1). This finding was interpreted as physiologic hydronephrosis related to pregnancy. Given the absence of concerning clinical or laboratory findings, the patient was managed conservatively with analgesia and observation, and her symptoms improved without further intervention.

Several years later, the patient re-presented with recurrent right-sided abdominal pain. The pain was described as intermittent and localized to the right lower quadrant, again raising concern for appendicitis. She denied associated gastrointestinal or urinary symptoms. Physical examination demonstrated mild right lower quadrant tenderness without peritoneal signs. Laboratory studies, including complete blood count and inflammatory markers, remained within normal limits. A contrast-enhanced CT scan of the abdomen and pelvis was obtained to evaluate for appendicitis. The appendix appeared normal, and no acute intra-abdominal or gynecologic pathology was identified. The patient was discharged with symptomatic treatment, and no definitive diagnosis was established at that time.

After an additional interval, the patient presented once more with worsening urinary symptoms, including dysuria, right flank pain, and lower abdominal discomfort. She denied fever, chills, nausea, or vomiting. Urinalysis demonstrated irritative changes without significant bacteriuria, and serum renal function tests were within normal limits. Given the recurrent nature of her symptoms and prior imaging findings, a contrast-enhanced CT scan of the abdomen and pelvis was performed to evaluate the urinary tract more thoroughly.

**Figure 2:**
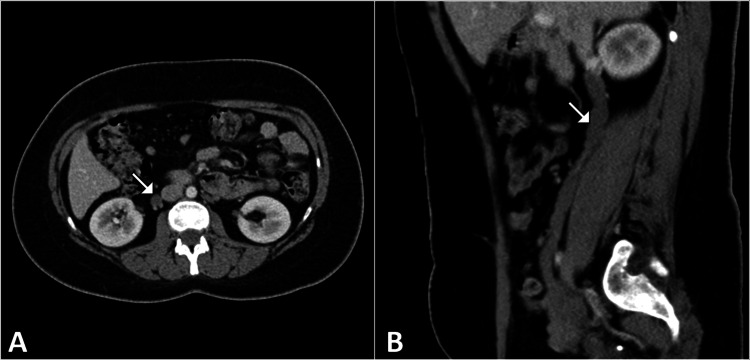
CT images demonstrating right ovarian vein dilatation causing ureteral compression Axial (A) and sagittal (B) contrast-enhanced CT images of the abdomen show a tortuous, dilated right ovarian vein (arrow) originating from the right renal vein and coursing anterior to the right ureter. The ureter is compressed between the dilated vein and the psoas muscle, resulting in upstream hydronephrosis.

CT imaging revealed a markedly dilated right gonadal (ovarian) vein measuring approximately 9 mm in diameter, draining into the right renal vein. The dilated vein coursed anterior to the right ureter, resulting in extrinsic compression and associated right-sided hydronephrosis (Figure 2). No ureteral calculi, intrinsic ureteral lesions, or mass lesions were identified. The appendix appeared normal, and no gynecologic or other intra-abdominal pathology was detected. Review of prior imaging suggested that the right-sided hydronephrosis identified during pregnancy may have represented an early manifestation of this condition rather than purely a physiologic change.

Management options were discussed in a multidisciplinary setting. Endovascular ovarian vein embolization via interventional radiology was considered a definitive treatment option. However, given the patient’s stable renal function, absence of severe or progressive obstruction, and intermittent nature of symptoms, conservative management was elected. This included analgesia, hydration, monitoring of renal function, and close outpatient follow-up.

## Discussion

OVS is a rare and often underrecognized cause of ureteral obstruction resulting from extrinsic compression by a dilated ovarian vein [1,3]. First described in the mid-20th century, the condition remained contentious for decades but is now increasingly accepted as an anatomic and clinical entity, particularly in women presenting with unexplained right-sided abdominal or flank pain and ipsilateral hydroureteronephrosis [1-4]. The pathophysiology of OVS relates to dilation and engorgement of the ovarian vein, most commonly on the right side, leading to direct compression of the adjacent ureter as it courses over the pelvic brim [3-5]. This venous enlargement may arise from variant embryological anatomy, hormonal influences, multiparity, and physiologic changes such as pregnancy, which transiently increases venous flow and diameter [1,5,6]. Importantly, while pregnancy-related ovarian vein dilatation is common, persistent compression severe enough to produce symptomatic obstruction appears to require a combination of venous enlargement and connective tissue fixation, making the ureter more susceptible to sustained extrinsic pressure [1-4].

Clinically, OVS presents with nonspecific symptoms that overlap with more common conditions, complicating diagnosis. Patients frequently report intermittent flank or lower abdominal pain, urinary symptoms such as dysuria or frequency, and recurrent UTIs, often in the absence of overt infection on culture. Hydronephrosis on imaging may be initially attributed to physiologic causes, for example, gestational hydronephrosis during pregnancy, delaying recognition of underlying venous compression. In the present case, mild hydronephrosis detected during pregnancy was initially considered physiologic, a common pitfall given the high prevalence of benign hydronephrosis in this population.

Imaging plays a central role in the diagnosis of OVS [4,5]. While ultrasound is often the first modality used and can reveal hydronephrosis and, occasionally, ovarian vein dilatation, CT with contrast provides superior delineation of vascular anatomy and its relationship to the ureter. Diagnostic criteria include a dilated ovarian vein with a diameter exceeding typical venous dimensions (often >8 mm) and evidence of ipsilateral hydroureteronephrosis without intrinsic ureteral pathology [3,4]. CT imaging in this patient during a subsequent presentation clearly demonstrated a markedly dilated right gonadal vein crossing anterior to and compressing the ureter, findings that are classic for OVS and enabled diagnostic confirmation [3,4].

The differential diagnosis of unilateral hydronephrosis and flank pain is broad, encompassing ureterolithiasis, intrinsic ureteral strictures, retroperitoneal fibrosis, gynecologic masses, and vascular compression syndromes such as nutcracker syndrome [1-5]. A systematic approach integrating clinical history, laboratory data, and imaging, including venous phases when indicated, is essential to exclude these entities. In particular, contrast-enhanced CT allows assessment of vascular structures, enabling identification of extrinsic compressive lesions that might be missed on ultrasound alone.

Management of OVS remains individualized, with options including conservative management, endovascular intervention, and surgical ligation [5,6]. Conservative treatment, comprising analgesia, hydration, and careful clinical or imaging follow-up, is appropriate for patients with mild, intermittent symptoms and preserved renal function. Case series and expert opinion suggest that this approach can be sufficient in select patients and that intervention may be deferred until symptoms become more severe or progressive obstruction is evident.

Endovascular ovarian vein embolization has emerged as a minimally invasive alternative to open or laparoscopic surgery, with technical success rates exceeding 90% and improvement in symptoms reported in a majority of cases [5,6]. Embolization induces thrombosis of the dilated vein, reducing venous pressure and relieving compression of the ureter. While data specific to OVS are limited, extrapolation from the pelvic venous disorder literature suggests that embolization can achieve significant symptomatic relief with low complication rates [5,6]. Laparoscopic ovarian vein ligation with ureterolysis is another well-described option, particularly for patients who are refractory to embolization or in whom endovascular access is not feasible; this approach allows direct decompression of the ureter and has demonstrated durable symptom resolution in published series [1-5].

## Conclusions

In this case, conservative management was selected due to intermittent symptoms, stable renal function, and absence of severe obstruction. This approach aligns with current practice for patients with mild or nonprogressive presentations, where the risks of intervention may outweigh the potential benefits. Continued follow-up is essential to monitor for changes in renal drainage or symptom burden that might warrant reconsideration of interventional therapy.
